# Symptoms and ECG changes precede sudden cardiac death in hypertrophic cardiomyopathy—A nationwide study among the young in Sweden

**DOI:** 10.1371/journal.pone.0273567

**Published:** 2022-09-14

**Authors:** Erik Börjesson, Bodil Svennblad, Aase Wisten, Mats Börjesson, Eva-Lena Stattin

**Affiliations:** 1 Sahlgrenska University Hospital/Östra Göteborg, Göteborg, Sweden; 2 Department of Surgical Sciences, Uppsala University, Uppsala, Sweden; 3 Department of Community Medicine and Rehabilitation, Geriatric Medicine, Sunderby Research Unit, Umeå, Sweden; 4 Department of Molecular and Clinical Medicine, Sahlgrenska Academy, Göteborg University, Göteborg, Sweden; 5 Center for Health and Performance, Department of Food, Nutrition and Sport Science, Göteborg University, Göteborg, Sweden; 6 Department of Immunology, Genetics and Pathology, Uppsala University, Uppsala, Sweden; University of California, Davis, UNITED STATES

## Abstract

**Background:**

Hypertrophic cardiomyopathy (HCM) is a major cause of sudden cardiac death (SCD) in the young. We aimed to characterize detailed family history, symptoms, hospital utilization and ECG changes before SCD.

**Methods:**

We extracted all cases suffering SCD with HCM from the SUDDY cohort, which includes all cases of SCD between 2000–2010 in Sweden among individuals aged 0–35 years along with their controls. We gathered data from mandatory national registries, autopsy reports, medical records, ECGs (including military conscripts), and detailed family history from an interview-based questionnaire (with relatives, post-mortem).

**Results:**

Thirty-eight cases (7 female), mean age 22 years, with HCM were identified. Among these, 71% presented with possible cardiac symptoms (chest pain [26%], syncope [22%], palpitations [37%]), before death; 69% received medical care (vs 21% in controls) within 180 days before death. The majority (68%) died during recreational activity (n = 14) or exercise/competitive sports (n = 12). Fifteen (39%) had a known cardiac disorder prior to death, with HCM being diagnosed pre-mortem in nine cases. 58% presented with abnormal ECG recordings pre-mortem, and 50% had a positive family history (1^st^-3^rd^ generation) for heart disease.

**Conclusion:**

In this comprehensive, nationwide study of SCD due to HCM, 87% (33/38) of cases had one or more abnormality prior to death, including cardiac symptoms, a positive family history, known cardiac disease or ECG abnormalities. They sought medical care prior death, to a larger extent than controls. These findings suggest that cardiac screening should be expanded beyond competitive athletes to aid SCD prevention in the young population with HCM.

## Introduction

Hypertrophic cardiomyopathy (HCM) is a genetic cardiovascular disease believed to affect one in 500 (0.2%) individuals in the general population [1]. In children, the prevalence is estimated to 2,9 per 100 000, and clinical diagnosis of HCM in childhood is much less frequent than in adults [2–4]. The clinical diagnosis of HCM is most often established by 2D echocardiographic imaging or cardiac magnetic resonance imaging and defined by increased left ventricular wall (LV) thickness, not solely explained by abnormal loading conditions (e.g., hypertension, valvular disease, congenital heart defects), with no prior assumptions regarding etiology or underlying myocardial pathology [5]. HCM is associated with increased morbidity and mortality, typically in the form of sudden cardiac death (SCD), often during exercise, though it often remains undiagnosed until death, as many experience few or no substantial symptoms [1]. In adults, the annual risk of SCD due to HCM is approximated to be 1.5–2% [5–7]. The risk of SCD in childhood has traditionally been thought to be much higher compared to adults [8], at around 4% annually [9]. In recent years lower SCD rates have been reported, around 1.5–2% annually [10, 11], while, O’Mahony *et al*. found an annual rate of 0.45% in adults, for the combination of SCD and appropriate ICD-shock [7]. It has been suggested that children between 8–16 years face the highest risk of SCD [12]. While SCD is rare, it is still a major cause of natural death in the young, with a huge impact on families and society. This is especially devastating since many of the individuals are young and sudden death is often the only manifestation of the disease [1, 13].

Extensive research has provided greater understanding of risk stratification of patients with HCM in general, with the recent European Society of Cardiology (ESC) guidelines suggesting assessment of clinical examination, family history, 48-hour electrocardiography (ECG), echocardiography and exercise test [5]. It is essential to identify at-risk patients to reduce the risk of SCD in young individuals with HCM, by exercise restriction, drug therapies and implantable cardioverter defibrillator (ICD) [5]. Cardiac screening of young competitive athletes has been proposed by the ESC [14], involving personal (symptoms) and family medical history (e.g., Premature death/HCM etc.), physical examination and resting 12-lead ECG. Indeed, a recent Danish study found that the majority of individuals suffering from SCD due to HCM had prior symptoms, and a vast majority also sought medical care prior to death, compared to controls [15]. This highlights the possibility of identifying at-risk individuals, and thus ultimately preventing death in some cases of HCM. In this study, we wanted to expand the current knowledge by adding ECG-evaluation and family history. There is a need for further studies in these patients to determine detailed clinical features for preventive purposes.

The aim of this study was to characterize clinical symptoms, medical history, family history and ECG findings before the occurrence of SCD caused by HCM, in young individuals from a comprehensive nationwide cohort.

## Methods

### Study design and study population

All individuals aged 0–35 years, suffering from SCD, between 2000–2010 in Sweden with autopsy findings of HCM (n = 38) were included in the study. We also included individuals who died suddenly and were diagnosed with HCM before death. The SUDDY database (The Swedish study of SUDden cardiac Death in the Young) comprises 903 individuals that died from SCD in Sweden aged 0–35, from January 1, 2000 until December 31, 2010, as well as 5 controls per case and parents of cases and controls. Of the 38 individuals in the present study, 35 were part of the SUDDY database [16].

### Control group

We used the Register of the Total Population and Population Changes (TPR), held at Statistics Sweden, to identify individuals making up the control group. For each case, five controls were identified, matched for gender, birth year, and county (geographical area of residency).

In addition, the parents of controls and cases were included, using the Swedish Multi-Generation Registry described below.

### Data sources used in the study

Every individual born in or living permanently in Sweden is given a unique 10-digit personal identification number (PIN). The PIN was used to link study subjects to different national registries, and for retrieval of data from medical records of SCD cases. For optimal identification SCD cases within the population, three registries were used:

-*the Swedish National Board of Forensic Medicine database (SFM)*, comprising all individuals who undergo a forensic autopsy in Sweden, a short medical background, disease codes and circumstances of death. We used the SFM registry to identify cases in our cohort and to retrieve data from the forensic reports;
- *the Cause of Death Registry (CDR)*, which includes death certificates issued by a physician containing details of the circumstances and underlying conditions leading to death. The CDR was used to identify cases in our cohort and to retrieve cause of death in deceased parents;
- *the National Patient Registry (NPR)* contains information on all in- and outpatient hospital activity, including international classification of diseases codes for each visit. The NPR was used to retrieve previously known diseases for cases, controls and parents. We also used the NPR to compare hospitalizations and outpatient visits prior to death between cases and controls.

### Forensics and clinical autopsy

The HCM diagnosis was defined either pre-mortem, in clinical setting from medical records, or post-mortem according to clinical or forensic autopsy. The forensic autopsy report contains a police report, information on examination of the body, results from histopathology, results from toxicology screen, and often interviews with witnesses and relatives as well as information from medical records. All forensic autopsies are performed or supervised by a limited number of experienced forensic pathologists, located in six regional departments. The clinical autopsy comprises examination of the body and results from histopathological investigations. Clinical autopsies are performed in numerous hospitals in Sweden according to several consensus documents and a Swedish accreditation standard protocol. Histological evaluations were performed in all subjects with forensic autopsy and in most of cases of clinical autopsy. HCM was mainly defined by the presence of increased Left Ventricular (LV) wall thickness that was not solely explained by abnormal loading conditions (e.g., hypertension, valvular disease, congenital heart defects) [5].

### Medical records

Medical records from visits recorded in the NPR were gathered. Information on symptoms, known disorders and ongoing medical surveillance, results from physiological and imaging studies and circumstances preceding the death were extracted.

### ECG recordings

All ECGs recorded at the time of conscription to military service were collected, as well as ECGs taken in connection to other medical visits, when available. Military conscription, for men, was mandatory in Sweden between 1901 and 2010.

### Interview-based questionnaire

In addition to reviewing death certificates, medical and autopsy reports, we gathered information about family history from an interview with first-degree relatives using a standardized questionnaire. The questionnaire contained items concerning medical information, family history, physical activity, food and stimulant habits, as well as prior symptoms. Questionnaires were conducted as a semi-structured telephone interview carried out by the authors. For each case, the closest family members of the deceased were contacted via mail with information about the study an invitation to participate.

## Definitions

### Definitions of sudden death, SCD and sports related death

*Sudden Death* was defined as a nontraumatic, unexpected fatal event occurring within 1 hour of the onset of symptoms in an apparently healthy subject. If death is not witnessed, the definition applies when the victim was in good health 24 hours before the event [17].

*Sudden Cardiac Death (SCD*) was defined as a congenital, or acquired, potentially fatal cardiac condition was known to be present during life; OR autopsy has identified a cardiac or vascular anomaly as the probable cause of the event; OR no obvious extra-cardiac causes have been identified by post-mortem examination and therefore an arrhythmic event is a likely cause of death [17, 18].

*Sports related sudden cardiac death* was defined as a “Nontraumatic SCD occurring within 1 hour of moderate- to high-intensity exercise in a competitive athlete”[19].

The deceased was considered an *athlete* if he or she did moderate- to high-intensity sports on a regular level and took part in competitions [20].

### Family and medical history

*A positive medical history* was defined as having known cardiac disease, other chronic disease or diagnoses that is associated with possible cardiac etiology.

*A positive family history*, indictive of the presence of cardiac conditions in relatives, was defined as follows: A positive first-degree history: an individual’s parents, siblings, and children; A positive second-degree history involves an individual’s grandparents, grandchildren, uncles, aunts, nephews, nieces, and half-siblings; A positive third-degree history include an individual’s great-grandparents, great grandchildren, great uncles/aunts, and first cousins.

### Symptom classification

*Cardiac symptoms* were defined as chest pain, angina, dyspnea, syncope, presyncope, chest discomfort, palpitations, autonomic symptoms.

*Diffuse cardiac symptoms* were defined as any of the following: nausea/vomiting, cold sweats, malaise, dizziness, chronic cough, and fatigue.

Symptoms occurring <24h before death were withdrawn from medical records and described as *prodromal symptoms* and possible cardiac.

*Other symptoms* were defined as symptoms occurring >24h before death.

### Electrocardiographic evaluation

In this study we used the “International recommendations for ECG interpretation in athletes”, by Sharma *et al*. (2017), to determine ECG findings that were considered abnormal [21].

Findings suggestive of HCM include left ventricular hypertrophy (LVH), T-wave inversion (TWI), ST-segment depression, pathological Q waves, conduction delays, left axis deviation (LAD) and left atrial enlargement (LAE).

Importantly, individuals that participate in regular intensive exercise over a long period of time often present with physiological adaptation to the heart with the presence of isolated left ventricular hypertrophy. These findings in an athlete are considered normal, and the athletic participation of all individuals was therefore considered in the evaluation of ECGs.

### Statistical methods

Descriptive statistical analysis was used to describe percentage of totals, mean, and interquartile range (IQR). To compare cases with controls, the reference date was defined for controls as the date when the control was of the same age as the corresponding case at death; for cases, the date of death. Conditional logistic regression was used to compare cases and controls. Statistical analyses were performed using Microsoft Excel and R, version 4.0.3. [22].

### Ethics

The study was approved by the Regional Ethical Review Board at Umeå University and later Uppsala University (Dnr: 2017/430 and 2017/431). Participating parents, spouses and siblings signed an informed consent form.

## Results

### Study population

A total of 38 individuals fulfilled the definitions of HCM and were included in the present study. Thirty-three cases were linked to NPR. Among the 38 included cases, three were not linked to the NPR due to lack of a PIN, and two cases were later reclassified as having HCM (from the unspecified cardiomyopathy group included in the SUDDY cohort).

### Clinical characteristics

The median age at the time of death was 22 years (range 0–35 years). There was a male predominance (81%; n = 31 male, 7 female). The median height and weight for the male cases were 184.0 centimeters and 76.0 kilograms, respectively [Table 1]. Height and weight were only recorded in half of the female cases.

**Table 1:** Baseline and clinical characteristics of young HCM cases suffering from SCD, in Sweden 2000–2010.

Characteristics	HCM cases (n = 38)
Median age (IQR) age at the time of death, years	22 years (IQR 14–28)
Male/Female gender, n (%)	31 (81); 7 (19)
Past medical history, n (%)	22 (58)
*•* Cardiac diagnosis (in total) *•* Other cardiac pathology *•* AVNRT *•* WPW *•* ASD *•* Atrial flutter *•* Unknown cardiomyopathy *•* Dilated cardiomyopathy	15 (39) 6 (16) 3 (8) 2 (5) 1 (3) 1 (3) 1 (3) 1 (3)
*•* HCM	9 (24)
Circumstances of death, n (%)	
*•* Sleep	4 (10)
*•* Rest	4 (10)
• Recreational activity (e.g., walking, gardening, sitting)	14 (37)
*•* Exercise	5 (13)
*•* Competitive sports	7 (18)
*•* Unknown	4 (10)
Witnessed deaths, n (%)	23 (61)
*•* CPR in witnessed/unwitnessed cases	23 (100); 7 (88)
Place of cardiac arrest, n (%)	
*•* In hospital death	11 (29)
*•* Out of hospital death	24 (63)
*•* At home	13 (34)
*•* Public place	11 (29)
*•* Unknown	3 (8)
Autopsy, n (%)	33 (84)
*•* Clinical autopsy	12 (29)
*•* Forensic autopsy	21 (55)

AVNRT; Atrial ventricular-nodal reentry tachycardia. WPW; Wolff-Parkinson-White. ASD; atrial septal defect.

Twenty-two (58%) of the 38 individuals had a previous known disease prior to death. Of the remaining cases, eight were considered healthy without previous disease and eight had no information available regarding previous disease. Fifteen (39%) of the 38 cases were diagnosed with a cardiac disorder, followed by psychiatric illness (n = 5) and asthma (n = 3) [Table 1]. Nine (24%) cases were diagnosed with HCM prior to death. The remaining six cases with a clinical cardiac diagnosis not considered to be HCM will be discussed in more detail in the “Family history” section below.

### Circumstances of death

Twenty-six individuals (68%) died during recreational activity (e.g., sitting, walking, gardening) (n = 14, 37%) or during exercise and/or competitive sports (n = 12, 31%). In seven of 38 (18%) cases, death occurred during competitive sports activity. Eleven (29%) died in hospital, 24 (63%) out of hospital and in four cases (8%), the place of death was unknown [Table 1].

Cardiac arrest was witnessed in 23 individuals and resuscitation was attempted in all of these cases. Resuscitation was performed in seven out of eight (88%) non-witnessed cases [Table 1]. In patients for whom cardiac arrest was witnessed, the most common rhythm encountered by prehospital personnel and emergency departments was ventricular fibrillation (VF; n = 11, 48%) and unknown rhythm (n = 11, 48%).

### Findings at autopsy

A vast majority of cases underwent autopsy after death (n = 33, 84%). Forensic autopsy was most often conducted (n = 21, 55%) followed by clinical autopsy (n = 12, 29%). In five cases, an autopsy was not conducted, and one case is unknown. In all the cases where an autopsy was not actively conducted, clinical diagnosis of HCM was previously established in the medical records.

The median heart weight for adult men were 253 g/m^2^ (168–557 g/m^2^) and for adult women 280 g/m^2^ (143–446g/m^2^). The median LV thickness for men and women was 19.5 mm (7-35mm) and 14.5 mm (10-23mm), respectively. Median septal thickness was 25mm (10-35mm) in men and 24mm (15-25mm) in women [Table 2]. A total of 22 toxicology screens were performed. A positive toxicology screen was seen in eight (36%) cases. One case had a toxicology profile positive for anabolic steroids, which the forensic pathologist concluded could have contributed to death. Nontoxic levels of alcohol were found in either blood or urine in nine cases but could not explain death in any of the cases.

**Table 2:** Autopsy findings among 33 SCD victims due to HCM.

Autopsy finding	
Autopsy type (n = 33)	Number (%)
*•* Forensic autopsy	21 (55%)
*•* Clinical autopsy	12 (29%)
Heart size/measurements (n)	Mean (min-max)
*•* Heart weight gr (33) 0-2years (5) 10–18 years (6) 20–35 years (22)	452.1 (27–865) 75.3 (27–130) 395.5 (180–865) 553.2 (324–833)
*•* Heart Weight g/m2 (25) 0–2 years (5) 10–18 years (5) 20–35 years (15) *•* Septum mm (14) ≥ 15 mm (12) *•* LV wall thickness mm (28) 0–2 years (4) 10–35 years (24)	191.2 (103–283) 296.8 (143–446) 274.0 (168–557) 23 (10–30) 18.9 (7–35) 11.0 (7–14) 20.2 (10–35)
*•* RV wall thickness mm (21)	8.0 (3–25)
0–2 years (3) 10–35 years (18)	5.3 (5–6) 8.4 (3–25)

SCD = Sudden cardiac death, HCM = hypertrophic cardiomyopathy, LV = left ventricle

RV = right ventricle.

### Family history

A positive family history, gathered from medical records (first to third degree), suggestive of heart disease was reported in 15 family members; two had no family history and 20 showed an unknown family history for SCD or HCM [Table 2]. The other six cases had cardiac disorders as follows: three cases with unknown cardiac pathologies; one case with atrioventricular nodal reentry tachycardia (AVNRT) and Wolff-Parkinson-White syndrome (WPW); one with atrial septal defect (ASD), atrial flutter, unknown cardiomyopathy, suspected WPW; and one with suggested dilated cardiomyopathy.

To complement medical records, additional information regarding family history was added by family interview in 22 cases; in 15 (n = 15, 69%) of those, a positive family history for cardiac disease/SCD was seen [Table 2]. The majority (n = 13, 59%) of cases interviewed presented with a positive family history for SCD in the first generation, followed by 2^nd^ (n = 6) and 3^rd^ (n = 5), respectively.

In summary, a positive family history for cardiac disease was seen in 15 cases collected from medical records and 15 from interviews. In six cases, the interview provided information about family history that was not known from medical records. This gives a total of 21 cases of positive family history, from medical records and interviews combined. This means that the family interviews provided 40% additional information regarding family history, compared to medical records alone.

### Cardiac symptoms

Data retrieved from medical records showed that 27 (71%) cases experienced possible cardiac symptoms prior to death. Data from interviews with relatives indicated that eleven (50%), experienced symptoms such as fatigue, dyspnea, palpitations, presyncope or syncope prior to death and eight out of 22 (36%) gave indications to people surrounding them that “something was not right”. Nine cases recorded one cardiac symptom, nine had two cardiac symptoms and nine had three cardiac symptoms or more. The most common cardiac symptom recorded was dyspnea (n = 13, 48%), followed by diffuse symptoms (n = 12, 44%; e.g., dizziness, fatigue, malaise), and then chest pain and palpitations, (n = 10, 37%) or both.

Prodromal symptoms <1 hour prior to death were recorded in 14 of 38 (37%) cases. Nausea/vomiting was the most common prodromal symptom, seen in five cases (13%).

### Electrocardiographic findings

In total, 28 individuals underwent ECG evaluation at some point during their lifetime. Of these, 23 (82%) had ECG findings that were considered abnormal. Twelve cases underwent ECG at the time of their conscription, and half (n = 6, 50%) of these were considered abnormal.

The mean age at which a pathological ECG was recorded was 17 years. The average survival from the moment a pathological ECG was recorded was 5 years. In the medical record data, thirteen individuals presented with possible cardiac symptoms before a pathological ECG was recorded and six individuals presented with an abnormal ECG before possible cardiac symptoms [Tables 3 and 4].

**Table 3:** Summary of abnormal ECG findings prior to death in young HCM individuals suffering from SCD.

Collected ECG	n = 28			
	Normal	5		
	Pathological	23		
Conscription ECG	n = 12			
	Normal	6		
	Pathological*	6		
Afflicted case (sex)	First pathological ECG	Pathological ECG age (years)	Time: pathological ECG to death (years)	Symptoms ECG-findings
Male	LAD, LVH, RVH	1	2	-
Female	ST-dep, V3-V5	2	8	-
Female	LVH, T-neg V1-V5, II	7	3	Before
Male	IRBBB, T-neg V1, V2, V4R, ST-dep, AvF, II, V6	11	1	Before
Female	LVH. Pat Q-wave V6	1	12	Before
Male	RBBB, Preexcitation (delta wave), LVH	8	6	Before
Male	AF, VT?	14	-	Before
Male	RVH, T-neg lat	-	-	-
Male	Wide QRS, high amplitude, ST-dep, left precordial leads RBBB?	10	7	Before
Male	ST-depression avR, II, III, AvF, V5-V6, IRBBB	17	4	After
Male *	RVH, RAD, ST-depression II, III, AvF	17	5	After
Male *	LVH, T-neg V3-V6, AvL	17	5	Before
Female	LVH V2-V6, ST-depression V5-V6	18	4	Before
Male	PSVT 151, Wide QRS, VT?	22	1	Before
Male *	Intraventricular block, LVH	18	6	After
Male	T-neg V1, V3-V5, AvF, III, LBBB?	25	-	Before
Male *	Pat Q-wave II, III, AvL, RAD, LVH	18	9	Before
Male *	LVH	18	10	After
Male	Pat Q-wave inf, III, AvF, Weak R-progression ant, leads	28	2	Before
Male	LVH	18	13	Before
Male	LVH, Deep V1, High V5	33	-	-
Female	Pat Q-wave III, LBBB	15	19	After
Male *	ST-dep, AvL, III, LVH	20	16	After

LAD, Left Axis Deviation; RAD, Right Axis Deviation; LVH, Left Ventricle Hypertrophy; LBBB, Left Bundle Branch Block; RBBB, Right Bundle Branch Block; AF, Atrial Fibrillation; VT, Ventricular Tachycardia; IRBBB, Incomplete Right Bundle Branch Block; PSVT, Paroxysmal Supraventricular Tachycardia; Dep, Depression; Ant, Anterior; Lat, Lateral; Inf, Inferior.

* Pathological ECG at conscription.

**Table 4:** Summary of abnormal findings prior to death in young HCM individuals suffering from SCD.

Case	Sex (male/female)	Age–pathological ECG	Abnormal ECG	Prior symptoms	Family history of cardiovascular disease ^a^	Positive Medical history	Any positive
1	M	-	-	-	-	-	✗
2	M	-	-	-	✓	-	✓
3	F	-	-	-	-	-	✗
4	M	-	-	✓	-	✓	✓
5	M	1	✓	✓	✓	✓	✓
6	F	2	✓	-	✗	✓	✓
7	F	7	✓	✓	✓	-	✓
8	M	11	✓	✓	✓	-	✓
9	F	1	✓	✓	✗	✓	✓
10	M	8	✓	✓	✓	✓	✓
11	M	14	✓	✓	-	-	✓
12	M	16	✓	-	✓	✓	✓
13	M	10	✓	✓	✓	✓	✓
14	F	-	✗	✓	-	✓	✓
15	M	-	-	✓	✓	✗	✓
16	M	-	-	-	✓	-	✓
17	M	17	✓	✓	✗	✓	✓
18	M	17	✓	✓	✓	-	✓
19	M	17	✓	✓	✓	✗	✓
20	F	18	✓	✓	-	✓	✓
21	M	22	✓	✓	✓	✓	✓
22	M	-	✗	-	✓	✗	✓
23	M	-	-	✓	-	✓	✓
24	M	-	✗	-	✓	✗	✓
25	M	18	✓	✓	✓	✓	✓
26	M	25	✓	✓	-	✓	✓
27	M	18	✓	✓	✓	✓	✓
28	M	-	-	-	-	✓	✓
29	M	-	✗	-	✓	✗	✓
30	M	18	✓	✓	-	✗	✓
31	M	-	-	✓	-	✗	✓
32	M	28	✓	✓	✓	✓	✓
33	M	18	✓	✓	-	✓	✓
34	M	33	✓	-	✓	✓	✓
35	M	-	-	✓	-	✓	✓
36	F	15	✓	✓	✓	✓	✓
37	M	-	✗	✓	✓	✗	✓
38	M	20	✓	✓	-	✓	✓

Tick, indicates a yes-answer; Cross, indicates a no-answer; Grey rows indicate cases with a pre-mortem HCM diagnoses

^a^ Information collected from both medical records and interview-based questionnaire.

### Contact with the healthcare system

A total of 32 cases were linked to the NPR, including information on all in- and outpatient hospital activity, as previously mentioned. A majority (n = 22, 69%) of these cases were hospitalized and/or had an outpatient visit 180 days prior to death, compared to 33/123 (21%) in the control group (p < 0.001). In the case group, healthcare was sought either at the hospital or outpatient, at both 180 and 90 days prior to death (69% and 55%, respectively; Table 4).

### Medications

Ten patients had ongoing treatment with beta-blockers, five with asthma medications, three with antidepressants, four with antiarrhythmic medications (incl. disopyramide), and one had treatment with a calcium channel blocker.

## Discussion

The present study showed that 71% of young individuals (aged 0–35 years) suffering SCD from HCM, in a nationwide Swedish cohort 2000–2010, presented with possible cardiac symptoms prior to death. The majority (69%) sought medical care within 180 days prior to death. We reviewed medical records, autopsy reports, ECG recordings and conducted a family survey to be able to describe prior symptoms, family and medical history, and details on any contact with healthcare before death. In addition, we found that over half of the cases presented with an abnormal ECG recording prior to death, and more than half of the cases had a positive family history for heart disease.

### Prevalence and histopathology

The SUDDY cohort includes 903 individuals aged 0-35-years that died of SCD between 2000–2010 in Sweden. A total of 38 individuals fulfilled the definitions of HCM, based on medical records and clinical and forensic autopsy, were included in this study. In 2021, the Association for European Cardiovascular Pathology released guidelines for the routine autopsy practice in the post-mortem investigation of cardiac hypertrophy at autopsy [23]. At the time of this study, no such consensus guidelines were established. Therefore, we can assume that different classification methods/definitions were used in different regions for the post-mortem diagnosis of HCM. This in turn might have resulted in over/underdiagnosing of individuals presenting with findings consistent with LVH, for example.

### Clinical characteristics

Clinical characterization of all young cases that died of SCD due to HCM demonstrated that a majority of cases occurred in ages 15–35, most commonly between 15–25 years. The overwhelming majority of cases were male (81%). A male predominance has been shown in many previous studies and the causes for this are not entirely clear, but it has been suggested to be attributable to differences in the participation rate, extent and intensity of physical exercise between sexes [24]. Male sex itself has been suggested to be a risk factor for SCD in adult HCM [24, 25]. However, in childhood HCM, age does not appear to be an independent risk factor for SCD, with evenly distributed events among sexes under the age of 15. After the age of 15 a clear age-related male preponderance appears, reaching the values seen in adults [12]. It has been suggested that children between 8–16 years face the highest risk of SCD [12]. Several risk-scoring systems are available to evaluate the risk of SCD in individuals and the need for prophylactic measures, e.g., ICD-placement [10, 11, 26]. A Swedish population-based study by Östman-Smith *et al*. (2010) looked at ECG features from 87 HCM patients and assessed the risk of SCD. Limb-lead amplitudes provided the best ECG-screening tool for high-risk HCM patients, especially in childhood HCM [26]. Recent studies investigating the validity of this ECG risk score [27, 28], compared to others, demonstrate conflicting findings, highlighting the ongoing debate regarding its merit in assessing children with HCM.

Thirty-one percent of cases died during or immediately after exercise and competitive sports, and an additional 37% of cases during recreational activities (e.g., gardening and walking). This could indicate that physical exertion, and its resulting physical and endocrine stresses on the heart, may act as a trigger for malignant arrhythmias, resulting in cardiac arrest in these individuals [29]. An autopsy-based study conducted by Weissler-Snir, Allan *et al*. (2019) concluded that 64.8% of SCDs due to HCM occurred during rest or sleep and 14.8% during moderate or vigorous exercise. Exercise-related SCD was negatively associated with age, and the risk of SCD during exercise was highest in individuals <20 years [30]. A 2011 study by Harmon, Asif *et al*. investigated the incidence of SCD in National Collegiate Athletic Association Athletes (NCAA) and found that SCD rates in the general population may be similar to athletes, and that SCD is three times more likely in black individuals than white [31]. A study by Dejgaard, Haland *et al*. (2011) found no association with exercise and the risk of ventricular arrythmias [32].

A positive toxicology screen was seen in eight out of 22 (36%) cases. One case had a toxicology profile including anabolic steroids, which the forensic pathologist concluded could have contributed to death. A low level of alcohol was found in either blood or urine in nine cases but could not explain death in any of the cases. Many cases tested positive for either alcohol, proarrhythmogenic or psychopharmacological substances, which all possess arrhythmogenic properties. Thus, it cannot be ruled out that these substances contributed to the development of malignant arrhythmias and possibly death.

### Cardiac symptoms and ECG findings

Our findings are in line with those of the Danish study by Winkel *et al*, which found that 74% of cases had documented possible cardiac symptoms prior to SCD [15]. We found very similar figures, showing that 71% had possible cardiac symptoms prior to death, based on data from medical records and family surveys.

Nearly 70% of cases sought medical attention within 180 days prior to death. A majority of those who underwent an ECG-evaluation at some point during their life showed abnormal findings. A 2004 study by Wisten *et al*. examined SCD from 1992–2000 and found 82% of ECGs were pathological in the HCM-group, 76% showed cardiac symptoms prior to death and 18% had a family history of cardiac pathology suggesting cardiomyopathy [33]. When comparing pre-mortem vs. post-mortem diagnosed cases of HCM, we concluded that those diagnosed pre-mortem presented with abnormal ECG recordings in all cases. In addition, the majority had prior cardiac symptoms and family history of cardiac disease. When looking at only the post-mortem diagnosed cases, we found that a majority also presented with cardiac symptoms and almost 50% had an abnormal ECG and previous disease suggestive of cardiomyopathy. Over 80% of the post-mortem diagnosed cases presented with one or more of the risk factors mentioned above.

Importantly, a common resting 12-lead ECG can identify most of the abnormalities associated with or suggestive of disorders linked with increased risk of SCD [34]. Therefore, ECG is recommended as part of cardiac screening in young, competitive athletes [14]. Symptoms did not correspond with an abnormal ECG in only one case. Out of the six cases that presented with an abnormal ECG prior to cardiac symptoms, four (67%) were found during military conscription. This shows that conscription may identify underlying HCM early in young individuals and may be a complement to cardiac screening of competitive athletes.

With the help of early symptoms, ECG interpretation, and family history it is possible to identify individuals at risk of SCD before a major adverse event occurs. Therefore, we suggest the following:

1. *Cardiac screening*. Both the European Society of Cardiology and the American Heart Association agree on the benefit of cardiac screening of athletes. As a result, a decline in SCD among athletes has been shown in studies [29, 35]. We previously showed an approximately 50% decline in SCD among young competitive athletes in the 2000s compared to 1992–99 [35], and suggested that cardiac screening, public awareness, and increased safety measures may have all contributed to this decline [35]. As of today, no recommendations are in place for screening non-athletes. Noteworthy, a recent policy statement from the American Academy of Pediatrics [36] highlights the possible role of the primary physician in screening non-athletic children. Many children are engaged in sports, sometimes in a competitive setting or at school, which makes it more difficult to determine who might be eligible for screening or not.

2. *ECG for everyone experiencing cardiac symptoms and chest discomfort* (not only during military conscription), has the potential to detect more cases of HCM. In all cases, in our study, where individuals experienced possible cardiac symptoms prior to SCD, an abnormal ECG was detected. This indicates that ECG remains a very powerful tool when investigating cardiac symptoms, especially in HCM. Many individuals had cardiac symptoms years before they were investigated, possibly indicating that these symptoms were not interpreted as cardiac. Only one of the seven cases with a suspected or clinically established asthma diagnosis was subsequently confirmed by a spirometry test. The majority (n = 5) of these cases were treated with asthma medications and had experienced symptoms such as breathlessness on exertion or dyspnea prior to death. It is certainly possible that many of the symptoms presented were not signs of asthma, but rather an insidious presentation of cardiomyopathy that was subsequently missed. With increased knowledge regarding possible cardiac symptoms, family history and basic 12-lead ECG, it may be possible to identify more of these patients at an earlier stage. Another way would be to have an ECG screening mandatory for every child, even in the absence of symptoms, at one point during their studies. This would also ensure that males and females have equal chances of being detected. Since many of the cardiac symptoms, especially the more common ones (e.g., fatigue, malaise, dizziness), are of insidious character and could easily be disregarded as other common non-cardiac symptoms found in the general population of this age group it might be difficult to raise suspicion based on possible cardiac symptoms alone.

3. *Extensive evaluation for individuals with a positive family history*. Family survey not only gave us information regarding symptoms prior to death, it also provided valuable information regarding medical and family history. In thirteen cases, individuals had a first-degree relative diagnosed with HCM; in six cases, a second-degree relative, and in five cases, a third-degree relative. In total, 15 cases had a positive first, second and third generation family history of HCM. Much of the potentially relevant family history, especially from second- and third-degree relatives, was not included in medical records.

### Strengths and limitations of the study

This nationwide cohort comprises data from different registries providing complete coverage of all SCD in Sweden. The completeness is unique and provides true incidence figures. The detailed family history collected from medical records and from relatives, as well as the presence of ECG recordings, provide unique possibilities to study SCD in young HCM patients.

Even though, inevitably, all information had to be collected retrospectively, this might cause an over-interpretation of possible cardiac symptoms when reviewing medical records as well as interviewing relatives (recall bias). Many of these symptoms presented as “possible cardiac” are common in the general population and might not reflect cardiac disease. Clinical and forensic autopsy diagnoses of HCM may also be incorrect in some cases, due to phenotypic and histological overlap with other cardiac disorders. Additionally, because the study is retrospective, we cannot be sure that pathological examinations were conducted uniformly at the different forensic departments responsible for performing the autopsies. The ‘sudden’ criteria might have been missed in some subjects. Data from hospital and outpatient visits were collected, from both cases and controls, at 3 and 6 months prior to death, where 9/15 (39%) had a known cardiac disease and nine had a pre-mortem diagnosis of HCM. Data is lacking regarding how many of these visits were the result of symptoms or planned follow-up of already diagnosed HCM.

In some cases, individuals fulfilled the definition of a competitive athlete, where you would expect a heart naturally adapted to physical exercise and findings such as left ventricular hypertrophy on ECG evaluation. Without additional family history, fitness status and other investigations, such as echocardiography, it can be difficult to differentiate whether the ECG findings can be considered pathological or physiological. In this study, we attempted to account for this using the “International recommendations for electrocardiographic interpretation in athletes” by Sharma *et al*. 2017 [21]. However, we cannot be certain that all ECGs were correctly categorized.

## Conclusions

In this nationwide cohort study, we confirmed and expanded on previous findings by showing a high frequency of cardiac symptoms prior to death in individuals who died of SCD due to HCM. The majority of cases who underwent ECG prior to death had abnormal recordings, nearly half of the cases had a positive family history for heart disease, and more than half of the cases sought medical attention in the months prior to death. These findings suggest it might be possible to predict, and possibly prevent, SCD in young individuals with HCM. Improved strategies to detect those at high risk of SCD must be considered in this endeavor. ECG screening should possibly be expanded beyond professional athletes and a routine school screening program may be the way to ensure equal detection among male and females. We also recommend including ECG at an earlier age in cascade-screening of individuals with family history of HCM.
